# Beyond the Needle: Transforming Traditional Healthcare with Microneedle Drug Delivery Systems

**DOI:** 10.3390/pharmaceutics18101274

**Published:** 2026-10-08

**Authors:** Arjun Gokulan Manivannan, Narayanan Jayasankar, Vivekanand Ankush Kashid, Ravish J. Patel, Bhupendra G. Prajapati

**Affiliations:** 1 Department of Pharmacology, SRM College of Pharmacy, Faculty of Medicine and Health Sciences, SRM Institute of Science and Technology, Kattankulathur, Chengalpattu 603203, Tamil Nadu, India; arjungokul2000@gmail.com; 2 MABD Institute of Pharmaceutical Education and Research, Babhulgaon, Yeola, Nashik 423401, Maharashtra, India; 3 Centre for Research Impact and Outcome, Chitkara College of Pharmacy, Chitkara University, Rajpura 140401, Punjab, India; 4 Ramanbhai Patel College of Pharmacy, CHARUSAT Campus Charotar University of Science and Technology, Anand 388421, Gujarat, India; 5 Department of Pharmaceutics, Parul Institute of Pharmacy, Faculty of Pharmacy, Parul University, Waghodia, Vadodara 391760, Gujarat, India; 6 Department of Industrial Pharmacy, Faculty of Pharmacy, Silpakorn University, Nakhon Pathom 73000, Thailand

**Keywords:** microneedle, polymers, transdermal drug delivery systems, improved bioavailability

## Abstract

In the present era, modern drug delivery systems play a fundamental role in ensuring the systematic delivery of the drug into the body, reaching the desired targets. Across recent decades, the drug delivery system has evolved from simple oral and injectable formulations to interpolating with various developed techniques to fabricate innovative treatment approaches for safer, effective, and better patient compliance. Progressive techniques like nanoparticles, microparticles, liposomes, polymer-based systems, 3D printing, and biological carriers have been extensively combined with specifically targeted drug-delivery techniques across infectious, oncological, pulmonary, and neurological conditions. Microneedle transdermal patches have advanced in tandem. They have gained attention for their advancement to surpass the stratum corneum, minimal invasiveness, independent delivery, and patient compliance. This review provides a translational perspective by integrating MN materials and device engineering with drug-delivery performance, patient usability, clinical evidence, manufacturing scalability, and regulatory considerations to examine the progression of MN technologies from laboratory development toward real-world healthcare implementation. Microneedles are fabricated in different types, such as solid, coated, hollow, dissolving, and hydrogel-based, to enhance precision in dosing, better permeability, and suitability across diverse populations, and complex molecules are designed, such as proteins, peptides, hormones, vaccines, and cosmetic agents. Moreover, studies suggest MN are widely accepted by geriatric and pediatric groups, reduce anxiety, and significantly benefit individuals with trypanophobia, swallowing difficulties in the case of oral doses, and compromised venous action, in addition to breaking social inequity. MN-induced microchannels can transiently increase skin permeability and may facilitate sustained or controlled drug delivery depending on MN geometry, formulation, application conditions, skin site, and individual skin characteristics. The persistence of these microchannels and their safety profile are dependent on both device and formulation characteristics. MN is a pivotal part of the novel drug delivery system, addresses the complications arising due to conventional drug delivery, and proves to focus on the improvement of pharmacokinetic parameters, patient-centric therapy, target delivery, and real-world accessibility. Further transformation in the enhancement of MN technology can lead to clinically promising drug delivery systems for the management of long-term treatment and boost next-generation therapies.

## 1. Introduction

The drug delivery system (DDS) is acknowledged as a prime process in the administration of a drug or any therapeutic substance in the body. It is chiefly focused on the technique and the duration of the substance to accomplish the target process at the specific site. It is an approach to enhance the formulation, gauge the rate of release of the therapeutic agent, and assess its impact on the pharmacological target. The device used to administer the drug, especially in some cases that require the insertion of a device in the body, also becomes the eternal part of the DDS [1,2]. The conventional DDS is largely characterized by various systems of administration that involve oral, rectal, and parenteral (Intravenous (IV), Intramuscular (IM), Subcutaneous (SC), and Intra-arterial (IA)) routes, as well as transmucosal, transnasal, transdermal, and intraosseous infusion. The oral route of administration primarily deals with the intake of therapeutic substances through the oral cavity. The absorption of substances through this route is governed by the stomach and small intestine. It is one of the most convenient and desired routes of administration, conferring distinct strength in patient compliance, non-intrusiveness, feasibility, and financial viability in production, and it can be most suitable in the treatment of Gastrointestinal (GI) infections [2,3]. Though this system supplies benefits, it is also subjected to central barriers and influencing factors such as physiochemical, metabolic, and biochemical barriers. Several factors that involve physiochemical parameters are poor water solubility, membrane permeability, chemical stability, constant ionization, dissolution rate, salt form, protein binding, complex formation, and adsorption [4,5]. The acid in the stomach, mucosal permeability, GI tract solubility, efflux transporters (P-glycoprotein (P-gp), Breast Cancer Resistance Protein (BCRP), Multidrug Resistance-associated Proteins (MRPs)), metabolic enzymes (CYP3A4, peptide, lipase), GI transit time, surface area (stomach < intestine), thickness of mucous layer, tight junctions, local irritation, nausea, and some food effects represent major physiological barriers. Intestinal metabolism (the Irishman brush border intercellular), first-pass metabolism (intestine and hepatic), and membrane transporters (uptake and efflux) present the major drawbacks in the absorption of therapeutic activity through the oral route. Passive diffusion and active transport are the two main techniques that affect the distribution of therapeutic activity [6,7].

A topical DDS mediates the release of the active ingredient/drug through the skin. In this type of administration, formulations are designed in such a way that they are applied onto the superficial areas of the body, like the skin, hair, eyes, nose, and vagina. This method is considered as one of the favourable systems for its convenience, economic viability, and local delivery [4,5]. It contributes to enhancing bioavailability by eliminating the pass metabolism, easing administration, and being atraumatic and nonintrusive. It yields a greater physiological and pharmacological response with lower systemic toxicity. It eliminates the complication of gastric pH variation and plasma level fluctuation [6].

The stratum corneum is the outermost layer of the skin that acts as the protective shield against the entry of any particle through the skin; hence, it is one of the prominent barriers for the penetration of the drug into the skin. Therefore, it is one of the parameters to be highly considered during the formulation of the drug. The commonly cited ~500 Da rule refers primarily to passive permeation through an intact stratum corneum. Because MNs physically bypass or disrupt the stratum corneum, this limitation should not be directly generalized to MN-mediated drug delivery [7,8]. The nature of the skin is highly varied across different regions, and in conditions like psoriasis and eczema, the skin is highly affected; their penetration and permeability are primarily objected to, which is a significant obstacle during formulation. The major difficulties that arise during formulation are the selection of excipients, as it must prevent irritation, dryness, and hypersensitivity, and the structured formulation has to be patient-concordant [9,10].

The parenteral route of administration refers to the introduction of a drug to the body via injection; it escapes the GI tract and delivers the active agent into systemic circulation directly. The injections are generally in the form of a solution, suspension, or emulsion. The injections are primarily delivered through IV, SC, IM, or intraperitoneal (IP) routes. Intradermal is a common invasive method with rapid onset of action, 100% viability, and is best for people with unconsciousness [10,11]. Though with these major advantages, it has certain drawbacks. In the SC route, it is difficult to control absorption. In the case of IM injection, only a limited amount of drug can be injected. It also has the added risk of peptide degradation with complications in nerve injury, haematoma, and abscess. IV injection could lead to thrombophlebitis (trauma to veins) and immune reaction with proteins and peptides. In all of these, pain and irritation are the two common limitations [7,9].

The limitations associated with conventional drug delivery, including challenges related to bioavailability, solubility, stability, targeting, and controlled drug release, have driven the development of diverse novel drug-delivery platforms. These systems use different carrier architectures and formulation strategies to modify drug disposition, release behaviour, stability, and tissue exposure [12].

The delivery platforms summarized in Table S1 differ primarily in their carrier architecture and mechanism of drug incorporation. Nanoparticle-based systems rely on discrete particulate carriers, whereas vesicular systems use enclosed lipid or amphiphilic structures and polymer-based systems use polymeric matrices, particles, or devices to control drug incorporation and release. Microneedle-based systems differ from these conventional carrier platforms by using micron-scale structures to physically bypass or disrupt the stratum corneum and facilitate intradermal or transdermal delivery. These platforms should therefore not be considered interchangeable, as their drug-loading mechanisms, release behaviour, stability, manufacturing requirements, and biological interactions can differ substantially [13,14].

**Figure 1:**
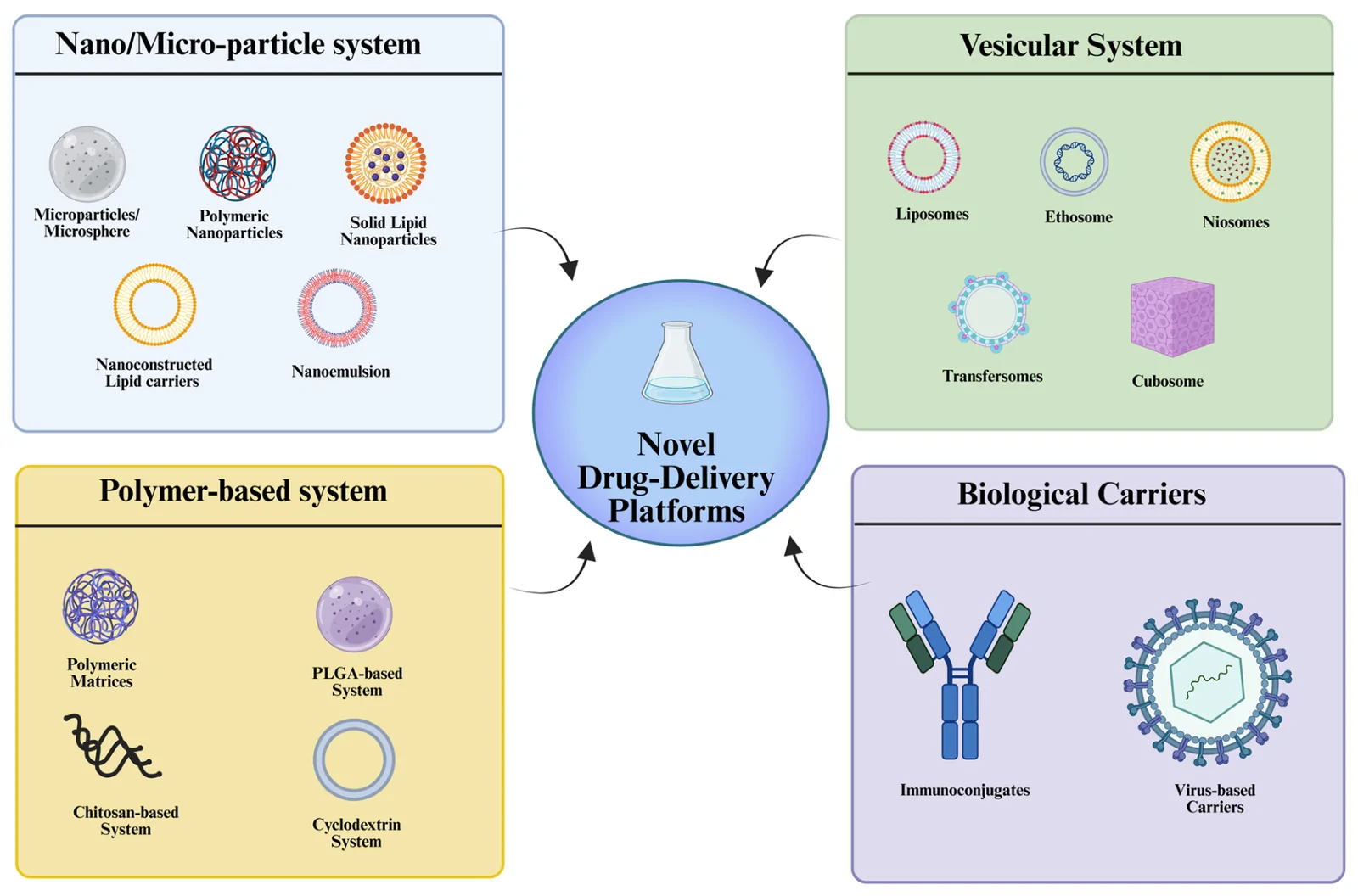
Overview of emerging drug-delivery platforms and their principal formulation strategies.

These advances in formulation and fabrication have expanded the range of strategies available for improving drug delivery, as illustrated in Figure 1. The approaches summarized in Table S2 illustrate that drug-delivery systems differ substantially in their mechanism of barrier interaction, payload characteristics, targeting strategy, invasiveness, and formulation requirements. Conventional and advanced carrier systems can modify drug stability, distribution, or release, whereas MN-based systems additionally provide a physical means of bypassing or disrupting the stratum corneum to facilitate intradermal or transdermal delivery. However, the advantages of MNs should be considered in relation to device-specific limitations, including mechanical integrity, insertion reproducibility, dose loading, formulation stability, manufacturing scalability, and patient usability [15,16].

Although several reviews have discussed microneedle (MN) fabrication, materials, therapeutic applications, and specific MN platforms, an integrated perspective connecting MN engineering with clinical and healthcare translation remains important. Therefore, this review aims to examine MN-based drug delivery from a translational perspective, linking material selection and device design with drug loading and release, skin interaction, patient usability, therapeutic applications, public-health relevance, clinical evidence, manufacturing scalability, and regulatory considerations. Particular emphasis is placed on the factors that influence the progression of MN technologies from laboratory and preclinical development toward clinical implementation. In contrast to reviews primarily focused on individual MN technologies, fabrication approaches, or specific applications, this review integrates the materials–device–drug delivery–patient–clinical translation pathway and critically discusses the major challenges that may influence reproducibility, usability, scalability, and real-world implementation of MN-based drug delivery systems.

Microneedles (MNs) have emerged as a minimally invasive approach for transdermal and intradermal drug delivery, with the potential to provide both local and systemic drug exposure. The MN DDS incorporates an array of submillimetre-sized needles (up to 1500 micrometres), which is facilitated using a base that is able to bypass the stratum corneum and transverse through the epidermis into the skin [8,17].

Microneedles (MNs) are minimally invasive drug-delivery devices consisting of arrays of micron-scale projections designed to interact with the skin barrier. Depending on their architecture, MNs can create transient pathways through the stratum corneum, deposit drug within the skin, or provide controlled access for fluid infusion or sampling. Different MN architectures—including solid, coated, hollow, dissolving, and hydrogel-forming systems—provide distinct mechanisms of drug incorporation and release. Their delivery performance depends on MN geometry, material properties, drug formulation, insertion conditions, skin characteristics, and the intended route of exposure. Accordingly, MNs should not be considered a single uniform delivery platform; each architecture presents specific advantages and limitations that influence its translational potential [17,18].

**Figure 2:**
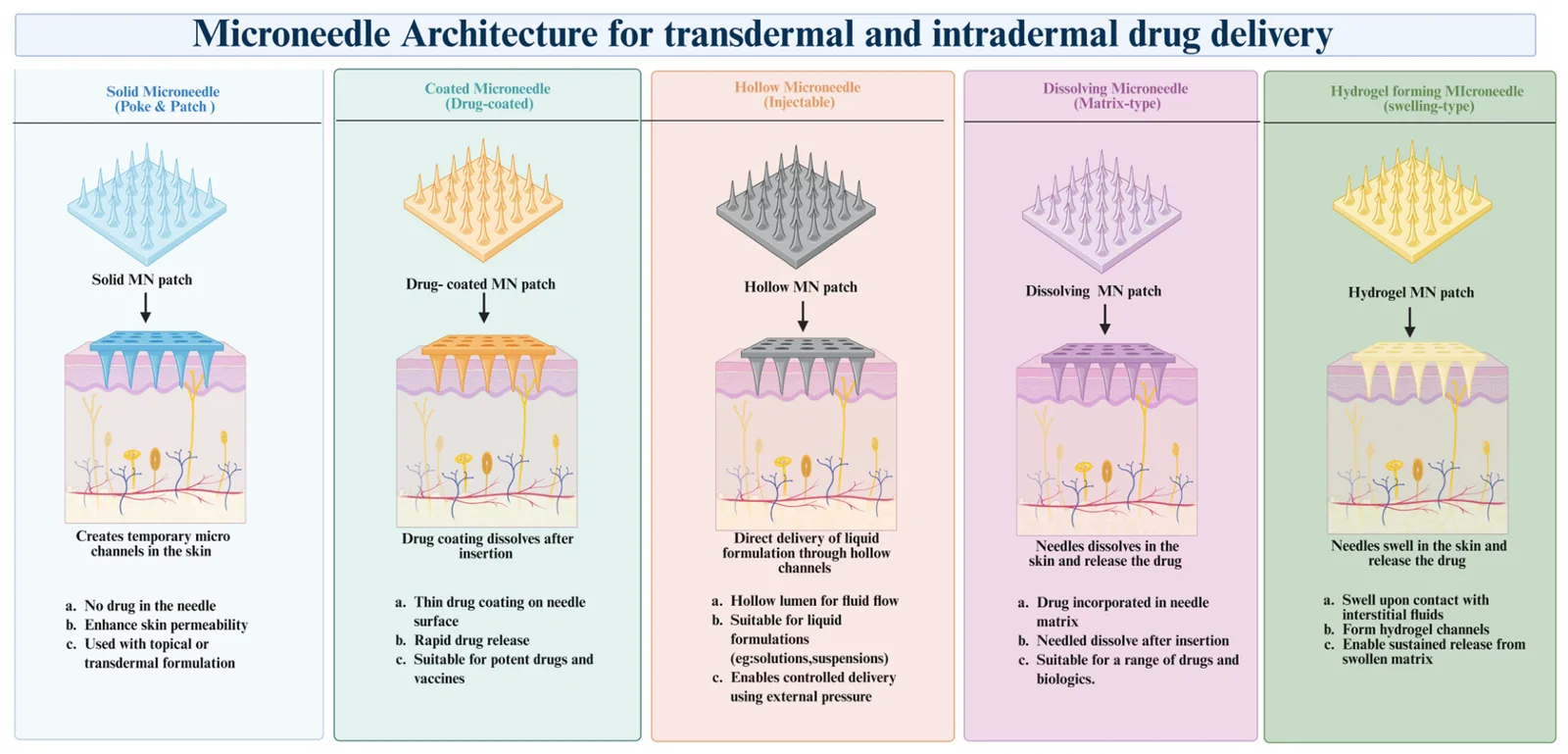
The schematic representation of different types of microneedle architecture and drug-delivery mechanisms.

The major MN architectures and their corresponding delivery mechanisms are summarized in Table S3 [19,20,21,22,23,24] and illustrated in Figure 2.

The five MN architectures differ primarily in how the therapeutic agent is incorporated, introduced into the skin, and subsequently released. Solid and coated MNs rely on either subsequent application of a drug formulation or deposition of the drug from the needle surface, whereas hollow MNs permit delivery of liquid formulations through an internal lumen. Dissolving MNs incorporate the therapeutic agent within a soluble or biodegradable matrix, while hydrogel-forming MNs typically establish a swollen pathway that facilitates diffusion from an associated drug reservoir. Consequently, differences in drug-loading capacity, mechanical strength, insertion behaviour, dissolution or swelling, dose control, and manufacturing complexity must be considered when selecting an MN architecture for a specific therapeutic application [25,26].

### Literature Search Strategy and Study Selection

This review was conducted as a narrative review to provide an integrated overview of microneedle (MN)-based drug delivery, including MN materials, device architectures, drug-delivery mechanisms, therapeutic applications, patient-related considerations, clinical translation, manufacturing, and regulatory challenges. Literature was identified through searches of PubMed and Google Scholar using combinations of relevant keywords and terms related to “microneedle,” “microneedle drug delivery,” “microneedle patch,” “dissolving microneedle,” “coated microneedle,” “hollow microneedle,” “hydrogel-forming microneedle,” “transdermal drug delivery,” “intradermal delivery,” “clinical translation,” “manufacturing,” “stability,” and “regulatory considerations.” The search covered literature across the available publication period without restricting the search to a specific starting year.

Studies were considered based on their relevance to the scope of the review, with particular attention to publications addressing MN materials and fabrication, device design, drug loading and release, skin interaction, therapeutic delivery, clinical or translational evaluation, manufacturing, stability, usability, and regulatory considerations. Relevant original research articles, clinical studies, review articles, and other appropriate scholarly sources were considered. Studies identified as unrelated to MN-based drug delivery or outside the scope of the review were excluded. Title and abstract screening were used to assess the relevance of retrieved literature before inclusion in the review.

As this article was designed as a narrative review rather than a systematic review or meta-analysis, literature selection and synthesis were based on thematic relevance to the objectives of the article rather than a formal systematic review protocol. Foundational studies were retained where necessary to describe established MN concepts, while recent literature was incorporated to reflect advances in materials, fabrication, clinical translation, multifunctional systems, manufacturing, and regulatory considerations.

## 2. How Microneedles Overcome Traditional Healthcare Limitations

Skin is structured in three layers: the outermost epidermis, the dermis and the hypodermis. To understand the MN mechanism and structuring, it is important to understand these structures. The epidermis, the outermost covering, is a strong barrier approximately 0.05–0.2 mm thick which stops the MN from entering. It has sublayers, with the stratum corneum being the outermost shield protecting the skin dermis from environmental hazards. MNs are fabricated to penetrate through the stratum corneum and reach the upper dermis layer. It provides effective drug delivery, averting the risk of deep penetration and preventing pain due to nerve contact [27,28]. To ease the insertion of MNs without any major cause damaging the skin, MNs are usually created in such a way that they perforate the corneum (~10 to 20 µm thick) and prevent the deformation of the needle. This approach enables localized delivery within the skin while limiting the depth of tissue penetration, although insertion depth and delivery performance depend on MN geometry, material properties, application force, and skin characteristics. The factors which may influence the insertion are stated below: geometry, material composition, skin properties, mechanical strength, insertional challenges, variability in skin elasticity, insertional-responsive MNs, implantable MNs, insertional force and pain, and skin interaction. These errors can be avoided by using insertional testing models, enhancement strategies and implicating bioinspired approaches [29,30,31]. MN addresses a great advantage in the paediatric and geriatric populations, offering an alternative to conventional dosage methods.

Heba Y. Raslan et.al. studied the parameters affecting the paediatric population regarding MN application. They also evaluated the potential advantages of MNs. They performed multiple interrogations among paediatricians, health care professionals and parents regarding concerns about the application of MNs. This study was a descriptive cross-sectional exploratory study conducted in Egypt during the period of November 2021 to March 2023. This study was conducted in two structures, one from the aspect of paediatricians and other in relevance to HCPs and parents. There were, in total, 154 paediatricians and 386 HCPs and parents reported. The study was completely based on questionnaires; there were different questions posed to both groups, focusing on the major challenges faced by the groups. The questions to the paediatricians mainly focused on the route of administration, constraints with the marketed paediatric doses and the class of drug needing the reformulation, whereas the other group included questions regarding the awareness, advantages and acceptance of MNs over conventional dosages. The data were gathered both online as well offline. The major challenge obtained in the group 1 study denoted unsuitable formulation, injection-related distress, and inadequate dose strength, whereas it solved the issues in administration of solid and liquid dosage. It was suggested that anti-infective, analgesic, anti-inflammatory, and cardiovascular disease drugs, as well as hormones and vitamins, could be best in the form of MN application. A questionnaire-based study involving healthcare professionals and parents evaluated perceptions of MN-based drug delivery for paediatric applications. The respondents reported potential advantages related to acceptability, pain, needle-related concerns, and treatment compliance. However, the study also identified concerns regarding emergency use, drug-loading capacity, and dose accuracy. Because the study assessed perceptions rather than therapeutic outcomes or pharmacokinetic performance in children, these findings should be interpreted as evidence of perceived acceptability and potential utility rather than clinical validation of MN-based paediatric drug delivery [9].

Megan N. Kelchen et al. studied the insertion of MNs in ageing skin. This study explored the transdermal delivery of a skin-impermeable drug by creating transient micropores that bypass the shielded function of the skin. It included 12 control subjects (25.6 ± 2.8 years) and 16 elderly test subjects (77.3 ± 6.8 years). The MNs were structured using four different geometrics, which differed in the length and number of MN. Array configurations were patterned in two forms. Ten MNs were arranged as 2 × 5 and 50 MNs were arranged as 5 × 10. Two insertions were performed (rotated 45°) to create 100 non-overlapping micropores for the 50 MN arrays. They were fabricated of stainless steel and engineered by Tech-Etch (Plymouth, MA, USA). The occlusive patches were fabricated using a rubber ring, a double impermeable polyester backing membrane, and double-sided medical tape. Tegaderm™ transparent dressings were utilized to retain the MN onto the skin. The MN and occlusive patches were carefully autoclaved preceding their application. The subjects were divided into four groups, whereas two of these groups included subjects exposed to the occlusion patches. Each subject was tested with two types of MNs that differed in their geometry at three different selective sites, respectively. Impedance spectroscopy was utilized to estimate disruption of the skin’s protective layer and its recovery immediately after application of an MN, whereas in the case of the non-occluded treated group, impedance measurement was done every 4 h during a total duration of 24 h, and in occluded groups, it was determined after 24, 48, 72, and 96 h. The occluded patches were replaced and inspected for redness, warmth, and oedema during every visit. The MN was also tested for its permeability, pore radius, time taken for pore closure, and the condition in the occluded and non-occluded groups. The results did not show any statistical divergence based on the difference in age or body mass index (BMI). In this study, MN application was well tolerated, and no adverse effects were observed during the reported observation period. Impedance value can be evidently declining, which is marked to produce the pore and enhance the disruption in the skin’s protective layer. Longer and an increased number of MNs showed better skin barrier impairment in elderly patients. The total permeability area simply defines the area presently available for the drug to travel. The total permeability area was delineated in elderly people compared to that of the control group, and a wide range of differences was also observed among the different geometries of MNs. The radius of the pore formed is smaller in the elderly compared to the control group, particularly when the density and needle size of the MN are larger. The pore closure was estimated to close on the accomplishment of the permeability of the MN. On comparing the conditions of the occlusion and non-occlusion patch, it was noted that in elderly subjects there was a delay in the pore closure or recovery of the skin barrier in the absence of the occlusion patch, whereas occlusion delayed pore closure and skin-barrier recovery during the observation period. These findings indicate that MN-induced skin-barrier disruption and pore recovery can vary with age, MN geometry, application conditions, and occlusion. In the elderly participants who were studied, differences in pore characteristics and barrier recovery were observed compared with younger controls, highlighting the importance of considering age-related changes in skin properties during MN design. The reported approximately 24 h persistence of detectable microchannels under the study conditions suggests a potential window for drug permeation; however, this observation should not be generalized to all MN systems or patient populations because pore persistence depends on device geometry, anatomical site, occlusion, application conditions, and individual skin characteristics. Although MNs may offer an alternative route when conventional administration is limited by factors such as swallowing difficulty or venous access, evidence for their broader acceptability, safety, and therapeutic effectiveness in older adults remains dependent on device-specific and clinical evaluation [11].

## 3. Self-Administration vs. Hospital Dependency

Self-administration is a potential advantage of selected MN systems because the patch format may simplify drug application outside conventional healthcare settings. However, the feasibility of self-administration depends on device design, dose control, application technique, drug stability, and the clinical context.

Jin et al. investigated a self-administered MN device based on an iontophoretic delivery principle for epinephrine administration. The study explored whether an integrated MN platform could provide an alternative approach for rapid, user-directed administration of epinephrine [12]. Their study defined a wearable epinephrine self-aid MN chip, which could ease the application of the drug. The chip consists of multiple layers of MN patch, drug-loaded connective hydrogel, iontophoretic electrodes, and a control circuit, overall encapsulated in 3D-printed housing with a spring lock mechanism. Analysing the devices, a polyvinyl alcohol–carboxymethyl cellulose (PVA-CMC) hydrogel solution was formulated and cast into polydimethylsiloxane (PDMS) MN moulds; freeze–thaw cycles were performed to assure its strength. The demoulded MN array was examined for its mechanical and skin penetration testing. COMSOL Multiphysics (v6.3) simulation was utilized to quantify the buckling and fracture force. A sulfobetaine-functionalized nanocellulose hydrogel solution was formulated, and epinephrine was embedded into the hydrogel. Subsequently, Zn^2+^ ions were added to potentiate the electrical conductivity of the gel. The hydrogel was maintained under the reported experimental temperature conditions; thereafter, Ag/AgCl electrodes were printed over the PI substrate and were charged using a coin battery. They were programmed over three current levels: low (0.1 mA/cm^2^), medium (0.3 mA/cm^2^), and high (0.5 mA/cm^2^). The device was subsequently evaluated for its ability to modulate epinephrine delivery at different electrical stimulation levels. The experimental findings indicated that the stimulation conditions influenced the delivery response, supporting the feasibility of integrating MNs with an iontophoretic system for adjustable drug administration. However, these findings primarily demonstrate device-level and experimental feasibility and should not be interpreted as evidence of established clinical efficacy or routine emergency use. The device was tested for in vitro and in vivo analysis. In vitro analysis was characterized using a Franz diffusion cell. Strat-M^®^ membrane or pig skin was employed to test the skin model. UV spectrophotometry was incorporated to quantify the permeation of epinephrine into the acceptor chamber, and the drug permeation was estimated based on the concentration time profile. COMSOL Multiphysics (v6.3) simulation was performed to test the combined effects of the electrical current and molecular diffusion on drug permeation through hydrogel and skin layers.

Hydrogel characterization was monitored using human embryonic kidney (HEK-293T) cell cultures. Calcein-acetoxymethyl ester staining and fluorescence microscopy were implemented to study the cell adhesion, morphology, and proliferation of the hydrogel surface. In vivo analysis was structured using a haemorrhagic shock model in Parma pigs. The mean arterial pressure was deduced using the loss of blood, and MN patches were applied onto the ear skin for penetration of the epinephrine to test under different electrical conditions. In accordance with conventional epinephrine injection, the heart rate and blood pressure were compared between both routes of administration. Hence, the efficacy of the device was analysed using the rate of change in haemodynamic parameters across low, medium, and high delivery modes. The result of this study stated that the buckling force of the MN decreased upon increasing their length during mechanical and COMSOL Multiphysics (v6.3) simulation assays. It ranged from 9.33 N at 600 µm to 5.25 N at 800 µm. The fracturing of the needle was screened using stress displacement testing, which revealed that a fracture was present at 341 µm upon applying a force of ~6.39 N per needle; hence, it was noted to be in the appropriate range, which ensured prevention of bending and breakage. It was observed that structured PVA-CMC-Na MNs had excellent mechanical strength and maintained uniformity in their geometry. Further formed micropores were tracked and confirmed healing within an hour of their formation, confirming their compatibility. An 8 s interval was observed between MN administration and drug permeation. On the determination of the electrical aspect of the devices, it was stated that increasing the Zn^2+^ concentration up to 10 mmol/L promoted conductivity, which was triggered due to imbalance in the ionic coordination and pore enlargement. It was also found that conductivity failed in cases, reasoning ionic aggregation and hydrogel disruption. The rate of drug delivery through preset electrical conductance was analysed by in vitro UV spectrophotometry, which suggested peak drug permeation at an interval of 5 min, followed by a decline that could be highly mediated by Ag/AgCl electrode oxidation. At the 15- and 90-min intervals, the cumulative rate of drug release was observed in high-, medium-, and low-grade model delivery. Hence, it was confirmed through COMSOL simulation, which was set to match the experimental results in delivering doses of 0.0814 mg (low), 0.353 mg (medium), and 1.04 mg (high) over an interval of 30 min. The in vivo haemorrhagic shock piglet’s method decoded that the low- and medium-grade models were discovered to increase the blood pressure at the module phase, whereas significant increases in blood pressure as well as heart rate were observed in the high-grade model, which could be compared to direct epinephrine injection. These findings indicated that the device response varied according to the programmed stimulation level. The higher stimulation condition produced a greater physiological response, whereas lower stimulation levels produced comparatively lower responses. Although these findings support the potential for adjustable dosing through electrical control, translation to emergency self-administration would require further evaluation of dose accuracy, pharmacokinetics, usability, device reliability, and safety in humans.

Self-administration represents a potential translational advantage of MN-based systems, particularly when conventional administration requires trained personnel or access to healthcare facilities. The epinephrine MN study demonstrates how device integration with electrical stimulation may provide an experimentally adjustable delivery approach. Nevertheless, the clinical value of such systems depends on reliable dose delivery, device robustness, drug stability, ease of use, and reproducibility across users. Therefore, self-administration should be considered a potential application of MN technology rather than a universally established replacement for healthcare-professional-administered treatment.

## 4. Reducing Cold-Chain Dependence for Vaccination

Cold-chain vaccines are specially designed formulations. From manufacturing to administration, these types of vaccines need recommended temperatures for their storage and transportation. The precise maintenance of vaccines is required to balance their potency. Hence, this highly structured vaccine comes along with a significant challenge. The first challenge rises in their development, as there is no reliable access to monitor their temperature system. Most of these vaccines are heat-sensitive and might get damaged upon freezing. Another major obstacle faced is the lack of appropriate storage and transportation equipment. This issue is mainly faced by low- and middle-income countries having problems of unreliable electricity and limited resources to operate refrigerators. Absorption refrigerators are highly used for their storage but require frequent maintenance and high fuel consumption. Insulated containers used for their transport make it difficult in conditions with high temperatures. Cold-chain vaccines require trained personnel for administration. Therefore, the lack of trained personnel is a constraint in their handling. Overall, the poor training raises the risk in storage and transportation [14].

A thermostable MN-based vaccine may offer an approach to reduce dependence on conventional cold-chain storage. This formulation could tackle the challenges raised by cold-chain vaccines; this could be an efficient formulation to optimize vaccine preparation, maintain their stability, and monitor their drying conditions. Self-application of this vaccine would also provide a reduction in the dependency on healthcare professionals and would also prove to be patient friendly [15].

Jessica C et al. designed a thermostable MN patch for the measles and rubella (MR) vaccine. The bivalent MN formulation demonstrated improved stability under selected non-cold-chain storage conditions, although stability varied with storage duration and temperature [16]. The current MR vaccine is conditioned to cold-chain storage, multiple-dose vials, lyophilization procedure, dependency on healthcare workers, precise reconstitution, and proper disposal. Hence, the designed MN patch for the MR vaccine could be an effective method to overcome all these drawbacks. This study involved the fabrication of the MR vaccine MN patch by optimizing the vaccine stock, following the casting of MNs. Development of the vaccine stock was done by obtaining the monovalent measles and rubella vaccine from the Serum Institute of India (Pune, India). The procured viruses were propagated in confluent Vero cell monolayers and were cultured in Dulbecco’s Modified Eagle Medium supplemented with 2% foetal bovine serum. They were incubated for 5 days at optimum conditions and were subjected to freeze–thaw cycles to release the intracellular viruses. They were further centrifuged to remove the debris. The clarified viral supernatants were collected and yielded titres of approximately 7 log_10_ TCID_50_/mL for measles and 5 log_10_ TCID_50_/mL for rubella. Finally, they were stored at −80 °C until further use. For the casting of the MN patch, the original concentration of the measles vaccine was incorporated, whereas using Vivaspin centrifugal filters with a 300 kDa molecular weight cut-off, the rubella vaccine was concentrated tenfold. The casting solution was processed by integrating the viral suspension, excipient, CMC, and buffer to maintain the pH and was further screened for its reliability and stability. Thereafter, PDMS moulds were used to design the MN patches. Each patch was precisely designed, comprising 100 conical MN having a height of 650 µm and distributed over 1 cm^2^. These moulds were designed in two layers; initially, 30 µL of the casting solution containing the vaccine was filled and dried under vacuum to prevent unfilled cavities. In case of the presence of excess formulation, it was removed. The second layer consisted of 28% *w/v* polyvinyl alcohol and 21% *w/v* sucrose and was dried for 2 days at room temperature using desiccant. Finally, the patches were demoulded and packed in aluminium pouches with a desiccant at various temperatures, including 5 °C, 25 °C, and 40 °C. The patches were alternatively stored at 5 °C and −20 °C for 1.5 h each for freeze–thaw analysis. Measles and rubella vaccine infectivity assays were performed concurrently with an in vivo immunogenicity study in seronegative juvenile rhesus macaques (2 years old, ~5 kg). They were selected with no prior exposure to measles or rubella. The MN applications were compared to the conventional subcutaneous injection and were randomly administered. The subcutaneous injections were administered at the back between the shoulder blades. The MN patch application involved the removal of hair using clippers and depilatory cream. The patches were applied using minimal pressure for 30 s and left undisturbed for 15 min to facilitate the dissolution of MNs. Serum analysis using the commercial ELISA kit was performed to quantify the serum IgG titres against measles and rubella. GraphPad Prism software (v10.3.1) was used to perform the statistical analysis with mean ± standard deviation with sample sizes of 2–4. The one-way ANOVA test was performed to analyse the buffer and freeze–thaw studies, whereas Student’s t-test was adapted to study the storage stability and IgG titres. The outcome of this analysis stated that the measles vaccine was stable during the drying process at a pH around 6.0 to 8.0, whereas the stability of the rubella vaccine was complicated; drying involved a lower pH, which concentrated the acids present, leading to damage in the rubella virus and lowering the infectivity. To ensure the best stability, the rubella vaccine was buffered at 7.5 pH. Among the various buffers tested, potassium phosphate maintained the stability of the vaccine and is also used in the current commercially available vaccine. Each excipient analysis involved the screening of 43 excipients. The excipients were analysed and were subjected to two conditions: one during the drying process and the other during storage at 40 °C. The rubella vaccine outperformed the measles vaccine in the tolerability of the excipients in the drying conditions. During drying, only a few excipients were able to maintain their potency in the measles vaccine; therefore, the excipients with stability in both the vaccines were selected for further assessment for their storage at 40 °C. It was identified that there were two different groups of excipients that maintained their potency in the drying conditions and storage. Asparagine, maltose, sorbitol, gluconate, and dextrose maintained their stability in the drying conditions, whereas during storage at 40 °C, histidine, sucrose, and trehalose maintained their potency; hence, it was considered that a combination of excipients would be able to optimize the stability better than the use of single excipients. There were four different combinations of excipients selected: histidine + sucrose, histidine + sodium gluconate, trehalose + asparagine, and sucrose + threonine. The sucrose–threonine combination excelled over the other selected combinations. The stability of the MN patch was tested for one-month, two-month, and three-month durations at 5 °C, 25 °C, and 40 °C. It was observed that there was no significant loss in the duration of one month, whereas in the interval of 2 months, the rubella vaccine showed loss in all the conditions, and at the end of 3 months, the measles vaccine was stable at 5 °C and 25 °C, but the stability declined at 40 °C. A freeze–thaw stability test was performed for three freeze–thaw cycles. It was found that there was no significant loss in the vaccine titres, and therefore the vaccine could also maintain its stability in the accidental freezing condition. The in vivo analysis in the races of macaques involved the comparison of MN patch administration against conventional subcutaneous injection; therefore, there was no significant difference found in the serum IgG antibody, and it was stated that the MN patch delivered immunogenic and effective results. The MN patches provided additional advantages compared with conventional subcutaneous injections, including needle-free administration, elimination of reconstitution, and reduced sharp waste. However, the stability findings indicate that the MN formulation may reduce dependence on conventional cold-chain storage rather than completely eliminate it. Therefore, the observed stability advantages should be interpreted as improved thermostability under specific storage conditions rather than evidence of complete controlled-temperature-chain independence.

## 5. Improved Bioavailability and Targeted Delivery

MN-based drug delivery may improve access to the skin and facilitate local or systemic delivery while potentially reducing some limitations associated with conventional administration. However, delivery performance remains strongly dependent on drug physicochemical properties, formulation composition, MN architecture, drug incorporation, release kinetics, skin permeation, and systemic absorption. Key formulation and pharmacokinetic considerations include drug loading and distribution within the MN matrix, aqueous solubility, stability during fabrication and storage, release behaviour, skin permeability, and variability in transdermal flux [13,32].

Successful MN-mediated delivery depends on reproducible insertion into the intended skin layer. Insertion performance is influenced by MN geometry, including tip and base dimensions, needle length, inter-needle spacing, material properties, and the force or velocity applied during administration. Geometry therefore represents a balance between penetration capability and mechanical integrity. Sharper and smaller tips may facilitate insertion but can also increase the risk of mechanical failure, whereas greater needle length may increase access to deeper skin regions but requires appropriate control of insertion depth. Inter-needle spacing is also important because closely packed arrays may experience the ‘bed-of-nails’ effect, in which deformation of the skin reduces the effective insertion of individual needles. Consequently, a single MN geometry cannot be assumed to provide equivalent delivery performance across different formulations, application methods, anatomical sites, or patient populations [33,34].

Biocompatibility assessment is an essential component of MN development because the device and its formulation interact directly with the skin and, depending on the design, may introduce materials or degradation products into the tissue. Evaluation should consider the intended duration and nature of contact and may include cytotoxicity, sensitization, irritation, and intracutaneous reactivity, together with additional assessments when prolonged exposure, systemic exposure, or specific material-related risks are anticipated. For biodegradable MNs, polymer degradation products and their local or systemic effects should also be considered. Thus, polymer selection requires a balance among mechanical performance, drug compatibility, degradation or dissolution behaviour, and biocompatibility [35,36]. If the contact lasts less than 24 h or between 24 and 30 h, the following tests, such as cytotoxicity, sensitization, skin irritation, and intracutaneous reactivity, are performed. Genotoxicity and subacute and chronic toxicity assays are undertaken in case the contact period is more than 30 h. These tests also regulate the harmful systemic and local effects. The choice of selection of polymer is highly attributed to safe degradation in the body and appropriate elimination while avoiding toxicity. Biodegradable and water-soluble polymers are frequently investigated for MN fabrication because they can provide different mechanisms of drug release and may reduce the need for removal of the inserted needle structure. Depending on the polymer and formulation, the incorporated drug may be released through polymer dissolution, swelling, diffusion, or degradation. However, controlled release should not be assumed solely from the use of a polymeric MN; release behaviour depends on polymer composition, molecular weight, crosslinking, drug–polymer interactions, MN geometry, and the surrounding skin environment. Drug loading and uniformity within the MN matrix also remain important considerations because insufficient or heterogeneous loading can affect dose reproducibility. Some polymeric MN formulations can be fabricated from aqueous or otherwise mild-processing conditions, which may be advantageous for heat-sensitive drugs [37,38,39]. However, the suitability of the fabrication process depends on the polymer, drug, drying conditions, and processing time; therefore, drug stability should be experimentally confirmed rather than inferred from the processing temperature alone. Stability assessment is necessary to determine whether the drug and MN structure maintain their intended quality during storage. Temperature, humidity, packaging, and moisture exposure can influence drug integrity as well as the mechanical and dissolution properties of the MN. Accelerated and long-term stability studies should therefore evaluate relevant quality attributes, including drug content, degradation, mechanical integrity, dissolution or insertion performance, and moisture uptake under appropriately justified storage conditions. For protein and other biologically sensitive therapeutics, the polymer matrix and formulation environment can influence molecular mobility, aggregation, and degradation during storage. Moisture management is particularly important because water uptake can alter polymer properties, mechanical strength, dissolution behaviour, and drug stability. Consequently, stabilizing excipients, appropriate packaging, and controlled storage conditions may be required depending on the specific drug–polymer combination. These formulation variables highlight that improved bioavailability through MN delivery is not determined by the device alone but by the interaction between drug properties, formulation composition, MN architecture, skin transport, and storage stability [8,40,41].

### Polymer Selection and Material-Dependent Performance

Polymer selection is a critical determinant of MN performance because polymer composition influences mechanical strength, water interaction, dissolution or degradation behaviour, drug compatibility, manufacturability, and storage stability. No single polymer provides optimal performance across all MN applications; therefore, material selection should be guided by the intended MN architecture, therapeutic payload, required release profile, fabrication process, and storage conditions [42,43].

Hyaluronic acid (HA) is a biocompatible, water-soluble polysaccharide that is particularly suitable for dissolving MNs because of its rapid hydration and dissolution. However, its high affinity for water can influence mechanical strength and moisture stability, requiring appropriate formulation and packaging strategies. Chitosan provides film-forming and mucoadhesive properties and can contribute to mechanical integrity, but its solubility and performance are influenced by molecular weight, degree of deacetylation, and formulation conditions. Gelatine offers biodegradability and favourable biological compatibility and can be processed under relatively mild conditions; however, its mechanical and storage properties can be affected by moisture and temperature [44,45,46,47].

Synthetic biodegradable polymers such as poly(lactic-co-glycolic acid) (PLGA), polylactic acid (PLA), and polycaprolactone (PCL) provide greater control over mechanical properties and degradation behaviour than many rapidly dissolving natural polymers. PLGA permits adjustment of degradation and drug-release behaviour through its composition and molecular characteristics, although degradation and processing conditions must be considered for sensitive payloads. PLA generally provides relatively high mechanical strength and slower degradation, which may be useful when structural persistence is required, but its limited water interaction can restrict rapid dissolution. PCL has favourable mechanical integrity and a relatively slow degradation rate, making it potentially useful for longer-term applications; however, its hydrophobicity can affect drug incorporation and release, particularly for hydrophilic compounds [48,49].

Silk fibroin represents another protein-based material of interest because its mechanical properties, crystallinity, and degradation behaviour can be modified through processing and formulation. Its use in MN systems has also been investigated for sensing applications, including glucose detection. However, silk-fibroin performance depends strongly on molecular structure, processing conditions, and crystallinity, and therefore should not be assumed to provide identical properties across different formulations [50,51].

Polymer selection involves a trade-off between mechanical strength, water sensitivity, dissolution or degradation rate, drug–polymer compatibility, manufacturability, and storage stability. Water-soluble materials such as HA may favour rapidly dissolving MNs, whereas PLGA, PLA, and PCL may provide greater structural persistence and controlled degradation. Natural polymers such as chitosan and gelatine offer biological compatibility but may require formulation strategies to improve mechanical and moisture stability. Silk fibroin provides an alternative protein-based platform whose properties can be tailored through processing. Consequently, polymer selection should be considered together with MN geometry, drug characteristics, fabrication method, packaging, and the intended clinical application rather than on the basis of biocompatibility alone [43,52].

## 6. Microneedles and Public Health: A Systemic Impact

From a public-health perspective, microneedle (MN) technologies may have implications for vaccination, treatment access, healthcare-resource requirements, biomedical waste management, and long-term disease monitoring. Large-scale immunization remains an important component of infectious-disease prevention, and MN-based vaccination has been investigated as an approach that could simplify administration and potentially improve acceptability. However, the magnitude of any population-level benefit depends on vaccine stability, dose accuracy, manufacturing capacity, delivery performance, cost, and implementation within existing healthcare systems [31,52].

MN-based vaccination may offer advantages related to administration without conventional hypodermic needles and may potentially reduce some logistical requirements associated with trained administration. These characteristics could be relevant to mass-vaccination programmes, particularly where healthcare-worker availability is limited. However, reductions in healthcare-worker requirements and improvements in vaccination coverage cannot be assumed solely from the MN format and would require evaluation in real-world implementation studies. Similarly, population-level effects on infection transmission, morbidity, or mortality would depend on actual vaccination coverage, vaccine effectiveness, and programme implementation rather than the delivery device alone [53].

Biomedical-waste management is another potential consideration. Depending on the MN design, the use of dissolving or biodegradable needle structures may reduce the generation of conventional sharp waste compared with reusable or disposable hypodermic devices [18]. However, the overall environmental benefit depends on the complete product system, including the backing layer, applicator, packaging, residual formulation, and disposal requirements. Therefore, reduced sharp waste should be considered a potential advantage of selected MN platforms rather than a universal property of all MN systems [17,54,55]. Limited access to healthcare professionals can contribute to disparities in treatment and preventive-care delivery. Self-administration or simplified administration of selected MN systems could potentially support healthcare delivery in settings where access to trained personnel is constrained [17]. Nevertheless, implementation in underserved populations would also require reliable product availability, appropriate training, affordability, storage, supply-chain infrastructure, and mechanisms for monitoring correct use [31,56]. For chronic non-communicable diseases requiring repeated monitoring, MN-based sensing platforms have also been investigated for measuring biomarkers such as glucose and other metabolites. Such systems could potentially support decentralized or home-based monitoring and reduce the need for some routine healthcare visits. However, their public-health value depends on analytical accuracy, calibration, device reliability, user adherence, data interpretation, and integration with clinical decision-making. Early detection or improved disease management therefore requires validation of the complete monitoring pathway rather than relying on the sensing capability of the MN device alone [31,36,56].

The potential public-health contribution of MN technology extends beyond the pharmacological advantages of transdermal delivery. Its possible relevance to vaccination, decentralized treatment, waste reduction, and biomarker monitoring suggests a broader healthcare role. Nevertheless, these benefits remain dependent on technological maturity, product stability, dose reproducibility, affordability, infrastructure, user training, and regulatory acceptance. Thus, MNs should be viewed as a platform with potential public-health applications rather than as a universally validated solution for healthcare-access challenges.

## 7. Clinical and Translational Evidence for Microneedle Systems

Clinical and translational studies provide important evidence regarding the feasibility, safety, usability, and therapeutic potential of MN-based systems. However, the available evidence encompasses different study types, including drug-delivery trials, diagnostic applications, local anaesthesia, cosmetic procedures, and early-stage investigational studies. Therefore, clinical evidence should be interpreted according to the study design, development stage, clinical objective, and reporting status rather than treating all MN applications as equivalent. Representative human and translational studies are summarized in Table S4 [57,58,59,60,61,62,63,64,65,66].

The studies summarized in Table S4 represent heterogeneous stages and applications of MN technology. Some investigations evaluate therapeutic drug delivery, whereas others examine local anaesthesia, diagnostic sampling, sweat induction, cosmetic applications, or device feasibility. Therefore, these studies should not be interpreted as equivalent evidence of clinical efficacy for MN-based drug delivery. In particular, registered studies without publicly reported results provide evidence of clinical investigation rather than demonstrated therapeutic benefit. The clinical maturity of each technology should consequently be considered in relation to study design, population, endpoint, regulatory status, and availability of peer-reviewed clinical outcomes.

## 8. Addressing Healthcare Inequity with Microneedles

The MN DDS fulfils the need of the healthcare system in the rural areas, which limits the necessary access to the clinical requirement. Conventional therapies require majorly trained professionals, sterile syringes, and clinical setups for proper guidance, which significantly fails in undeveloped areas. MN-based approaches break the huge barrier in the lack of resources, reaching widespread areas. Great MN drug delivery does not require huge setups or trained professionals and deals with minimal procedural protocol, reaching a widespread population.

It is also efficiently simpler in transportation, which is the pivotal concern in countries lacking the high-tech cold-chain maintenance in transportation. The dissolvable MN patch can be maintained at thermostability at varied temperatures. In the developing countries, its cost-effectiveness minimizes the dependency on refrigerators and limits the biomedical waste disposal [28,67].

MN drug delivery also provides relaxation of transportation to the elderly population and those dealing with chronic disorders. MN use restricts the requirement of frequent visits to the hospitals and improves patient compliance by reducing the use of painful invasive methods, enduring its vulnerable nature [18,68].

Universal health coverage is the core idea in promoting MN drug delivery, enabling access to essential medical coverage for the worldwide population. It requires varied approaches to meet the lack of infrastructure and workforce to overcome inequity [29].

## 9. Smart and Multifunctional Microneedle Systems

Recent advances have extended MN technology beyond conventional drug delivery toward integrated systems capable of combining therapeutic delivery with sensing or auxiliary physical functions. These systems represent an emerging area of MN research rather than an established component of routine MN-based drug therapy.

### 9.1. Mn-Integrated Sensing

MNs can access interstitial fluid and have therefore been investigated for minimally invasive monitoring of biomarkers such as glucose. For example, smartphone-controlled MN-based continuous glucose-monitoring systems and silk–fibroin MN platforms for visual glucose detection have demonstrated the potential of combining skin-accessible sampling with sensing technologies. These approaches may support minimally invasive biomarker monitoring; however, analytical accuracy, sensor stability, calibration, biofouling, device durability, and clinical validation remain important considerations before widespread clinical implementation [30,69].

### 9.2. Multifunctional Therapeutic Platforms

MN systems have also been combined with additional physical or electrical functions to create multifunctional platforms. A recent study described a self-powered MN patch integrating mild microwave thermal therapy and electrical stimulation for infected diabetic-wound management. Such systems illustrate how MNs may serve as an interface between localized delivery and auxiliary therapeutic modalities. However, these multifunctional platforms involve additional engineering and safety considerations, and their reported therapeutic effects should be interpreted according to the specific experimental model and device configuration [70,71,72].

### 9.3. Translational Perspective

The integration of sensing, stimulation, and drug delivery may provide opportunities for more personalized and responsive MN-based systems. Nevertheless, these technologies remain at different stages of development and should not be considered equivalent to clinically established MN drug-delivery products. Future studies should establish reproducible device performance, dose control, analytical accuracy, long-term stability, safety, usability, and clinical benefit before such multifunctional systems can be broadly translated into healthcare practice [72,73,74].

## 10. Challenges and the Path Forward

Microneedle-based drug delivery has demonstrated substantial potential for minimally invasive administration; however, translation from laboratory-scale development to reproducible clinical products remains dependent on several interconnected technical, manufacturing, quality, and regulatory considerations.

### 10.1. Manufacturing and Quality Control

Large-scale production requires reproducible fabrication of MNs with consistent geometry, mechanical integrity, drug content, and insertion performance. Critical quality attributes may include needle height and dimensions, fracture resistance, drug-loading capacity, content uniformity, dissolution or swelling behaviour, and skin-insertion performance. Manufacturing approaches such as mould-based fabrication and additive manufacturing may differ in scalability, dimensional reproducibility, production rate, and process control. Therefore, standardized manufacturing processes and validated quality-control methods are required to minimize batch-to-batch variability [74,75,76].

### 10.2. Dose Accuracy and Drug Uniformity

Dose delivery is influenced not only by the nominal drug loading but also by drug distribution within the MN array, formulation properties, insertion efficiency, and the amount of drug actually deposited into the skin. This is particularly important for coated and hollow MN systems, where coating uniformity, delivered volume, flow characteristics, and device operation can affect dose reproducibility. Accordingly, drug content, dose uniformity, insertion performance, and delivered-dose recovery should be evaluated as part of product characterization [51].

### 10.3. Mechanical and Insertion Performance

MNs must possess sufficient mechanical strength to penetrate the intended skin layer without unacceptable bending or fracture. At the same time, excessive mechanical strength or inappropriate geometry may influence insertion behaviour and tissue interaction. Insertion testing should therefore be conducted using validated experimental approaches appropriate to the device, with consideration of needle geometry, material properties, application force, insertion speed, and anatomical site. Reproducibility of insertion across different users and application conditions is also important for clinical translation [38,51].

### 10.4. Material and Formulation Safety

Selection of polymers and other materials requires consideration of biocompatibility, drug compatibility, dissolution or degradation behaviour, residual components, and stability during processing and storage. For MNs manufactured using additive manufacturing or photopolymerization approaches, residual monomers, photo initiators, or other process-related substances may require appropriate assessment. For biodegradable or dissolving systems, degradation products and their local or systemic effects should also be considered. Sterility or appropriate microbial-control strategies are particularly relevant for products intended to penetrate the skin [51,76].

### 10.5. Packaging and Stability

MN products may be sensitive to moisture, temperature, oxygen, and mechanical stress during storage and transportation. Packaging must therefore protect the MN structure and maintain drug quality throughout the intended shelf life. Stability studies should evaluate relevant attributes such as drug content, degradation, moisture uptake, mechanical integrity, insertion performance, dissolution or swelling behaviour, and microbial quality, as appropriate to the product. The interaction between the MN formulation and packaging material should also be considered during product development [38,51].

### 10.6. Applicator and Human Factors

Consistent administration may depend on the force, speed, angle, and duration of MN application. Variability in manual application can therefore influence insertion depth and delivered dose. Where an applicator is required, its reproducibility, usability, mechanical reliability, and compatibility with the MN patch should be evaluated. Human-factor assessment is particularly relevant for systems intended for self-administration because the final performance may depend on the user’s ability to correctly apply the device [51].

### 10.7. Regulatory and Clinical Translation

MN products may incorporate characteristics of a drug, medical device, biologic, or combination product depending on their composition, therapeutic function, and mode of action. Regulatory development therefore requires early consideration of product classification and appropriate quality, safety, efficacy, and performance requirements. Clinical translation should be supported by a progressive evidence package extending from material characterization and in vitro testing to mechanical and insertion assessment, biocompatibility, stability, preclinical studies, and appropriately designed human studies. The regulatory pathway will ultimately depend on the specific MN product and its intended clinical use [76,77,78].

Future development should therefore focus on establishing standardized manufacturing processes, validated quality attributes, reproducible dose delivery, appropriate material and microbial controls, robust packaging and stability strategies, reliable application systems, and product-specific regulatory pathways. Addressing these factors together may facilitate the transition of MN technologies from experimental platforms toward clinically reproducible drug-delivery products.

## 11. Conclusions

Microneedle (MN)-based drug delivery represents a versatile minimally invasive approach that can facilitate intradermal and transdermal administration of selected small molecules, macromolecules, vaccines, and other therapeutic agents. The reviewed evidence indicates that MN performance is strongly influenced by the interaction among material selection, device architecture, drug formulation, insertion characteristics, skin properties, and intended route of delivery. Different MN architectures provide distinct mechanisms for drug incorporation and release, and therefore their advantages and limitations should be considered on a device- and formulation-specific basis.

The available clinical and translational evidence demonstrates that MN technologies have progressed beyond laboratory fabrication into human studies involving drug delivery, local anaesthesia, vaccination-related applications, diagnostic monitoring, and other specialized uses. However, the evidence remains heterogeneous in terms of study design, clinical indication, development stage, and availability of clinical outcomes. Consequently, potential benefits such as improved acceptability, simplified administration, reduced dependence on conventional injection procedures, and enhanced delivery across the skin barrier should be interpreted according to the specific MN platform and supporting evidence rather than as universal properties of all MN systems.

Several challenges continue to influence clinical translation, including dose loading and uniformity, mechanical integrity, insertion reproducibility, skin-to-skin variability, drug and polymer compatibility, moisture sensitivity, storage stability, scalable manufacturing, sterility or microbial control, packaging, applicator performance, and regulatory classification. Multifunctional MN systems incorporating sensing, electrical stimulation, or other technologies further introduce additional requirements for analytical validation, device reliability, and safety. Addressing these challenges will require standardized quality attributes, reproducible manufacturing processes, appropriate preclinical evaluation, well-designed clinical studies, and product-specific regulatory strategies.

Future research should therefore move beyond demonstrating proof-of-concept fabrication toward establishing reproducible dose delivery, long-term stability, clinically relevant performance, usability, and scalable manufacturing. Integration of materials science, pharmaceutical formulation, device engineering, clinical pharmacology, manufacturing science, and regulatory expertise may support the responsible progression of MN technologies from experimental platforms toward clinically validated drug-delivery products.

## Data Availability

All the data that support the findings of this study are included in the manuscript. AI disclosure statement: During the preparation of this manuscript, we used a generative AI–based language assistance tool only to improve grammar, clarity, and readability of the text. The tool was applied for general language editing across the manuscript, including the abstract and main text. No AI was used for data analysis, interpretation, or generation of scientific content, and all content was reviewed and approved by the authors, who take full responsibility for the manuscript.

## Supplementary Materials

The following supporting information can be downloaded at: https://www.mdpi.com/article/10.3390/pharmaceutics18101274/s1, Table S1: Comparison of Different Drug-Delivery Platforms; Table S2: Comparison of Conventional and Advanced Drug-Delivery Approaches with Microneedle-Based Delivery; Table S3: Comparison of Microneedle Architectures for Drug Delivery; Table S4: Representative Clinical and Translational Studies Involving Microneedle-Based Systems.

## Abbreviations

DDS	Drug Delivery System
IV	Intravenous
PDMS	Polydimethylsiloxane
IM	Intramuscular
SC	Subcutaneous
IA	Intra-arterial
IP	Intraperitoneal
ECG	Electrocardiogram
GI	Gastrointestinal
RF	Radio Frequency
MN	Microneedle
COPD	Chronic Obstructive Pulmonary Disease
CTCL	Cutaneous T-cell Lymphoma
CNS	Central Nervous System
BBB	Blood–Brain Barrier
P-gp	P-glycoprotein
BCRP	Breast Cancer Resistance Protein
MRPs	Multidrug Resistance-associated Proteins
MLV	Multilamellar Vesicle
SUV	Small Unilamellar Vesicle
LUV	Large Unilamellar Vesicle
PVA-CMC	Polyvinyl Alcohol–Carboxymethyl Cellulose
HEK-293T	Human Embryonic Kidney Cell
MR	Measles and Rubella
EMLA	Eutectic Mixture of Local Anaesthetics
MNA-D	Micro Needle Array–Doxorubicin
BMI	Body Mass Index
ROS	Reactive Oxygen Species
