# Supplementary material for: Beyond the Needle: Transforming Traditional Healthcare with Microneedle Drug Delivery Systems
Source: Pharmaceutics. 2026 Oct 8;18(10):1274. doi: 10.3390/pharmaceutics18101274 (PMC13708118; doi:10.3390/pharmaceutics18101274)
Supplement: Supplementary file 1 [file pharmaceutics-18-01274-s001.zip › pharmaceutics-4540368-supplementary.pdf]

pharmaceutics

MDPI

Review

Beyond the Needle: Transforming Traditional Healthcare with Microneedle Drug Delivery Systems

Arjun Gokulan Manivannan [1], Narayanan Jayasankar [1,*], Vivekanand Ankush Kashid [2,3], Ravish J. Patel [4] and Bhupendra G. Prajapati [5,6,*]

1 Department of Pharmacology, SRM College of Pharmacy, Faculty of Medicine and Health Sciences, SRM Institute of Science and Technology, Kattankulathur, Chengalpattu 603203, Tamil Nadu, India; arjungokul2000@gmail.com
2 MABD Institute of Pharmaceutical Education and Research, Babhulgaon, Yeola, Nashik, 423401, MaharashtraIndia
3 Centre for Research Impact and Outcome, Chitkara College of Pharmacy, Chitkara University, Rajpura 140401, Punjab, India
4 Ramanbhai Patel College of Pharmacy, CHARUSAT Campus Charotar University of Science and Technology, Anand 388421 Gujarat, India
5 Department of Pharmaceutics, Parul Institute of Pharmacy, Faculty of Pharmacy, Parul University, Waghodia, Vadodara 391760, Gujarat, India
6 Department of Industrial Pharmacy, Faculty of Pharmacy, Silpakorn University, Nakhon Pathom 73000, Thailand
* Correspondence: narayanj@srmist.edu.in (N.J.); bhupen27@gmail.com (B.G.P.)

Table S1. Comparison of Different Drug-Delivery Platforms.

S. No.	Drug-delivery platform	Representative systems/approaches	Primary delivery role	Key consideration	Reference(s)
1	**Nano/microparticle systems**	Microparticles/microspheres; polymeric nanoparticles; solid lipid nanoparticles; nanostructured lipid carriers; microemulsions/nanoemulsions; selected inorganic nanoparticles	Encapsulation or incorporation of therapeutic agents within particulate carriers to modify solubility, stability, distribution, or release	Particle size, surface properties, drug-loading capacity, stability, biological fate, and manufacturing influence delivery performance	[2,4,13]
2	**Vesicular systems**	Liposomes, including MLVs, SUVs and LUVs; ethosomes; niosomes; virosomes; cochleates; cubosomes; transfersomes	Encapsulation and transport of therapeutic agents using lipid- or amphiphile-based vesicular structures	Vesicle composition, membrane properties, drug–carrier interactions, stability, and release behaviour determine performance	[2,5,6]

3	**Polymer-based systems**	Natural and synthetic polymeric nanoparticles; chitosan- and albumin-based systems; PLGA-based systems; cyclodextrin-containing formulations; polymeric microspheres and devices based on PLA, PLGA, or PGA	Controlled, sustained, or targeted delivery through polymer-mediated encapsulation, matrix formation, or degradation	Polymer selection affects drug compatibility, degradation/dissolution, release kinetics, mechanical properties, biocompatibility, and manufacturability	[7,27,35]
4	**Biological carriers**	Immunoconjugates; virus-based carriers	Use of biological recognition or carrier mechanisms to facilitate selective delivery of therapeutic agents	Immunogenicity, biological safety, carrier stability, targeting specificity, manufacturing, and regulatory requirements	[2,37]
5	**Beaded drug-delivery systems**	Drug-containing beads incorporated into dosage forms or capsules, including systems designed for acid-sensitive or sustained-release delivery	Modulation of drug release, including sustained or controlled release	Bead composition, drug loading, release kinetics, gastrointestinal stability, and dosage-form design influence performance	[2,27]
6	**Microneedle-based delivery**	Solid, coated, hollow, dissolving, hydrogel-forming, and hybrid microneedles	Physical disruption or bypass of the stratum corneum to facilitate intradermal or transdermal delivery	MN geometry, mechanical strength, drug loading, insertion reproducibility, dissolution/swelling, formulation stability, manufacturing scalability, and usability influence performance	[18,25,26,28]

Table S2. Comparison of Conventional and Advanced Drug-Delivery Approaches with Microneedle-Based Delivery.

S. No.	Delivery approach	Representative formulation/system	Barrier bypass / delivery mechanism	Payload / application	Targeting or delivery advantage	Invasiveness / acceptability	Key limitation	Reference(s)
1	Conventional oral delivery	Conventional oral dosage forms	Gastrointestinal absorption	Small molecules and other orally administered therapeutics	Convenient and widely established route	Non-invasive; generally high patient familiarity	Gastrointestinal degradation, variable absorption, first-pass metabolism, and food/drug interactions may affect exposure	[1–3]
2	Liposomal delivery	Amphotericin B liposomes	Carrier-mediated delivery through lipid vesicles	Antifungal therapy; leishmaniasis	Can modify drug distribution and formulation-related tolerability	Minimally invasive when administered parenterally	Vesicle stability, manufacturing complexity, and formulation-dependent drug loading	[2]

3	Targeted liposomal delivery	Sugar-grafted liposomes	Ligand-mediated targeting	Leishmaniasis	Potentially enhanced targeting and therapeutic exposure	Depends on route of administration	Targeting efficiency and biological variability require validation	[2]
4	Drug–antibody conjugates	Daunomycin conjugates	Antibody-mediated recognition of target-associated antigens	Cancer therapy	Potential for selective cytotoxic drug delivery	Generally, requires parenteral administration	Complex manufacture, immunogenicity considerations, and heterogeneous target expression	[2]
5	Stimuli-responsive delivery	Thermosensitive liposomes	Heat-triggered drug release	Paclitaxel; localized cancer therapy	Enables externally triggered release under appropriate conditions	Requires administration of the carrier and controlled stimulation	Requires appropriate stimulus and precise control of release conditions	[2,12]
6	Polymeric nanoparticle delivery	Polyvinylpyrrolidone–paclitaxel nanoparticles	Nanoparticle-mediated drug incorporation and tissue exposure	Tumour-targeted drug delivery	Can modify drug distribution and release	Usually requires administration of the formulation	Particle stability, drug loading, biological fate, and manufacturing considerations	[2,7,35]
7	Inhalation delivery	Iloprost inhalation formulation	Pulmonary deposition	Pulmonary arterial hypertension	Direct delivery to the respiratory tract with potential for prolonged local exposure	Non-invasive administration but requires inhalation technique	Patient technique, pulmonary deposition variability, and formulation/device dependence	[2]
8	Device-based delivery	Metered-dose inhaler	Device-assisted pulmonary delivery	Asthma and COPD	Direct pulmonary drug deposition	Non-invasive; dependent on correct device use	User technique and device/formulation performance can influence dose delivery	[2,30]
9	BBB-disruption approach	Mannitol-mediated BBB opening	Temporary modification of BBB permeability	CNS drug delivery	May facilitate access of therapeutic agents to the CNS	Invasive/clinically controlled procedure depending on implementation	Safety, reproducibility, and control of BBB opening require careful evaluation	[2]
10	Polymeric brain-targeting system	Hexapeptide-functionalized polymeric nanoparticles	Nanocarrier-mediated targeting	CNS drug delivery	Potentially enhanced CNS penetration	Administration-dependent	Targeting specificity and biological transport remain important limitations	[2,27]
11	Transdermal vesicular delivery	Bioadhesive/deformable liposomes	Interaction with and transport across skin barrier	Chronic disorders	Potential for localized or sustained transdermal delivery	Minimally invasive/non-invasive depending on	Skin barrier variability and formulation-dependent penetration	[2,25]

						formulation and application		
12	Transdermal reservoir system	Liposomal reservoir system	Localized deposition and extended release through skin	Benzocaine; local pain relief	Localized and potentially prolonged drug release	Non-invasive application	Skin permeability, drug loading, and release control may limit delivery	[2,35]
13	Microneedle-based delivery	Solid, coated, hollow, dissolving, and hydrogel-forming MNs	Physical disruption or bypass of the stratum corneum through micron-scale projections	Small molecules, proteins, peptides, vaccines, hormones, and other therapeutic agents	Can enhance intradermal/transdermal delivery while allowing different release and administration strategies depending on MN type	Minimally invasive; acceptability depends on device design, application method, and patient population	Dose loading, mechanical strength, insertion reproducibility, formulation stability, manufacturing scalability, and clinical validation remain important considerations	[8,9]

Table S3. Comparison of Microneedle Architectures for Drug Delivery.

S. No.	MN type	Delivery principle	Representative materials	Drug-loading / dose considerations	Mechanical / functional requirements	Key advantages	Key limitations / translational considerations	Typical applications	Reference(s)
1	Solid MN (poke-and-patch)	MNs create transient microchannels in the skin, followed by application of a drug-containing formulation	Silicon, metals, ceramics, polymers	Drug is generally supplied in a separate formulation; delivered dose depends on the applied formulation and reproducibility of insertion	Sufficient mechanical strength and reproducible insertion without fracture	Simple architecture; can enhance skin permeability	Requires two-step administration; insertion depth and subsequent drug application influence delivery reproducibility	Proteins, hormones, vaccines and other macromolecules	[21]
2	Coated MN (coat-and-poke)	Therapeutic agent is coated onto the MN surface and deposited during insertion	Metals, silicon, polymers and other coating-compatible materials	Loading is constrained by available needle surface area, coating thickness, formulation viscosity and drug stability	Adequate coating adhesion, uniformity, mechanical integrity and reproducible insertion	Direct deposition of drug into skin; relatively rapid release may be achieved for suitable formulations	Limited surface area can restrict drug loading; coating uniformity and drug stability require control	Vaccines, insulin, proteins and other biomolecules	[21]

3	Hollow MN (poke-and-flow)	Drug formulation is delivered through a lumen into the skin	Silicon, metals, polymers	Dose is governed by formulation concentration, delivered volume and flow control; there is no universal absence of dose limitation	Adequate wall strength, lumen patency, flow control and resistance to fracture/leakage	Can deliver liquid formulations and provide controlled infusion	Complex fabrication; clogging, leakage, fracture and larger tip dimensions may affect performance	Proteins, vaccines, nucleic-acid formulations and diagnostic applications	[21,22]
4	Dissolving MN (poke-and-dissolve)	Water-soluble or biodegradable MN matrix dissolves or disintegrates after insertion and releases the incorporated drug	Hyaluronic acid, PVP, PVA, CMC and other soluble/biodegradable polymers	Drug loading depends on polymer composition, drug concentration, MN volume and formulation stability; loading capacity is formulation-dependent rather than inherently "minimal"	Sufficient strength for insertion before dissolution and controlled dissolution after insertion	No need for needle removal; reduced sharp waste; suitable for incorporation of sensitive therapeutics depending on formulation	Limited loading capacity for some formulations, moisture sensitivity, mechanical-strength constraints and stability concerns	Vaccination, macromolecule delivery and dermatological/cosmetic applications	[21,23]
5	Hydrogel-forming MN (poke-and-release)	Swellable MNs form a hydrated pathway through which drug from an attached reservoir can diffuse into the skin	Crosslinked hydrophilic polymers and hydrogel-forming polymer networks	Dose depends on reservoir formulation, drug concentration, swelling behaviour and diffusion characteristics	Adequate insertion strength followed by predictable swelling and dimensional stability	Can provide prolonged delivery and allow separation of the drug reservoir from the MN matrix	Requires optimization of swelling, diffusion, mechanical properties, drug stability and device integration	Sustained delivery and selected biosensing/drug-delivery applications	[19,20,21,24]

Table S4. Representative Clinical and Translational Studies Involving Microneedle-Based Systems.

S. No.	Study / Application	MN Platform / Formulation	Study Type / Design	Population / Enrollment	Trial / Registry ID	Clinical Status	Primary Outcome / Endpoint	Reported Outcome / Publication Status	Reference(s)
1	Glucose Measurement Using Microneedle Patches (GUMP)	Microneedle patch using a biocompatible polymer or metal for	Human diagnostic / feasibility study	Human participants; enrollment details not reported in	Not reported in the cited source	Human clinical/diagnostic study; status not reported	Glucose measurement performance and patient	Comparable glucose measurements and reduced pain were	[57]

		glucose measurement		the cited manuscript source		in the cited source	acceptability compared with conventional methods	reported in the manuscript	
2	Randomized intra-individually controlled, double-blind trial comparing siSPARC MN patch with siSPARC + siLR4A MN patch for post-surgical scar appearance	Short siRNA-loaded microneedle patches	Phase 2; randomized, intra-individually controlled, double-blind clinical trial	Human participants; enrollment details not reported in the cited manuscript source	Not reported in the cited source	Phase 2 clinical study; results not yet published according to the manuscript	Post-surgical scar appearance	Outcomes not yet published according to the manuscript	[58]
3	Efficacy of Microneedle Patch on Topical Ointment Treatment of Psoriasis	Hyaluronic-acid-based dissolving MN containing calcipotriol–betamethasone	Human controlled clinical study	Patients with psoriasis; enrollment details not reported in the cited manuscript source	NCT02955576	Recruiting in the registry information reported in the literature; Phase: Not Applicable	Psoriasis severity, drug delivery/penetration, and adverse effects	Clinical comparison with ointment-only and normal-patch groups; study evaluated modified PASI and adverse effects	[59]
4	A Pilot Study to Compare the Efficacy of Pilocarpine Microneedles with Iontophoresis Method for Sweat Induction in Healthy Human Subjects	Solid water-soluble MN array containing pilocarpine	Human pilot / diagnostic study	Healthy human subjects; exact enrollment not reported in the cited manuscript source	NCT04732195	Completed; Phase: Not Applicable	Sweat production and sweat chloride concentration	Study evaluated MN-induced sweating against iontophoresis; subsequent review reports that safety/tolerability criteria were met but efficacy criteria were not met	[60]
5	A Study to Evaluate the Safety, Reactogenicity, and Acceptability of a Placebo Microneedle Patch in Healthy	Solid water-soluble conical MNs with hypoallergenic adhesive	Human safety, reactogenicity and acceptability study	Healthy infants and young children; exact enrollment not reported in the	Not reported in the cited source	Human clinical study; status not reported in the cited manuscript source	Safety, reactogenicity, acceptability and local skin reactions	The manuscript reports serious/grade 3 adverse events, skin irritation and new medical conditions; interpretation	[61]

	Infants and Young Children			cited manuscript source				should follow the original study report	
6	RF Microneedle Therapy Maintains the Treatment Effect of Melasma: A 32-week, Prospective, Randomized Split-face Study	Pulsed radiofrequency microneedle device	Prospective randomized split-face clinical study; 32-week follow-up	Human participants; exact enrollment not reported in the cited manuscript source	Not reported in the cited source	Completed human clinical study; publication available	Melasma pigmentation and post-treatment severity	Improvement in pigmentation/melasma severity was reported in the manuscript	[62]
7	Microneedle Intra-Arterial Injection With Pars Plana Vitrectomy for Acute Retinal Artery Occlusion	47-gauge intra-arterial MN for delivery of a thrombolytic agent into the retinal artery during vitrectomy	Prospective human interventional clinical study	24 participants; adults ≥18 years; acute retinal artery occlusion	NCT07151755	Recruiting; Phase: Not Applicable	Best-corrected visual acuity (BCVA; LogMAR) at 1 month	Clinical study ongoing; results not yet reported	[63]
8	The Use of MicronJet600 Microneedle for Intradermal Lidocaine Injection to Ensure Local Anaesthesia During Insertion of Peripheral Venous Catheters	MicronJet600 MN device delivering 100 μL lidocaine intradermally	Human clinical study	102 participants; healthy volunteers/patients	NCT05108714	Completed; Phase: Not Applicable; target enrollment 102	Pain reduction and effectiveness of local anaesthesia during peripheral venous catheter insertion	Lidocaine delivered using the MN device reduced cannulation-associated pain; the study was completed	[64]
9	Phase I, Single-Arm, Open-Label, Dose-Escalation Trial of Micro Needle Array-Doxorubicin (MNA-D) in Patients with Cutaneous T-cell Lymphoma	MN-array patch containing low-dose doxorubicin for intradermal chemotherapy	Phase I; single-arm, open-label, dose-escalation interventional study	20 participants; adults ≥18 years with Stage IA/IB cutaneous T-cell lymphoma	NCT02192021	Phase I; Completed according to later literature/registry-derived sources	Safety, tolerability and dose escalation of MNA-doxorubicin	Results were not available in the cited clinical-trial literature; later reviews report the study as completed	[65]
10	Transdermal Microneedle Patch to Enhance Topical	Maltose-based dissolving MN patch; 1 × 1 cm²,	Phase II; randomized, active-	20 participants; children with	NCT05078463	Completed; Phase II; study period	Pain/anaesthesia during venous	MN pretreatment improved anaesthesia/pain outcomes	[66]

Anaesthesia Before Intravenous Line Insertion for Blood Transfusion in Paediatric Thalassemia Patients	36 MNs/patch in the manuscript	controlled, crossover clinical study	thalassemia requiring IV cannulation		September 2021–August 2022	cannulation; skin-conductance and pain-related outcomes	compared with EMLA alone; clinical study completed
